# Nationwide trends in intensive care unit utilization in the elective endovascular treatment of unruptured intracranial aneurysms

**DOI:** 10.1177/15910199241233028

**Published:** 2024-03-07

**Authors:** Varun Padmanaban, William J. Benjamin, Austin Cohrs, Francis J. Jareczek, Sprague W. Hazard, Joseph Christopher Zacko, Ephraim W. Church, Scott D. Simon, Kevin M. Cockroft, Douglas L. Leslie, David Andrew Wilkinson

**Affiliations:** 1 Department of Neurosurgery, Pennsylvania State University College of Medicine, 12311 Penn State Milton S. Hershey Medical Center, Hershey, PA, USA; 2 21614 University of Michigan Medical School, Ann Arbor, MI, USA; 3 Center for Applied Studies in Health Economics, Pennsylvania State University College of Medicine, Penn State Milton S. Hershey Medical Center, Hershey, PA, USA; 4 Department of Anesthesia and Perioperative Services, Pennsylvania State University College of Medicine, 12311 Penn State Milton S. Hershey Medical Center, Hershey, PA, USA; 5 Department of Radiology, Pennsylvania State University College of Medicine, 12311 Penn State Milton S. Hershey Medical Center, Hershey, PA, USA

**Keywords:** Unruptured cerebral aneurysm, intracranial aneurysm, endovascular procedure, intensive care unit, cost-effectiveness, healthcare cost

## Abstract

**Objective:**

Multiple studies suggest routine post-operative intensive care unit (ICU) stays after endovascular treatment (EVT) of unruptured intracranial aneurysms (UIAs) is unnecessary, though rates of ICU utilization nationwide are unknown. We aim to evaluate rates and characteristics of ICU utilization in patients undergoing elective endovascular repair of UIAs.

**Methods:**

This is a retrospective cohort study utilizing a nationwide private-payer database in the United States to evaluate the ICU utilization in patients undergoing elective endovascular repair of UIAs between 2005 and 2019. Demographics and pre-operative comorbidities as well as post-procedural complications and discharge status were compared. An analysis of charges and costs was also performed.

**Results:**

Among 6218 patients who underwent elective EVT of a UIA, 4890 (78.6%) were admitted to the ICU post-operatively. There were no differences in age, sex, or Charlson comorbidity scores in patients admitted to the ICU post-operatively compared to those admitted elsewhere. ICU utilization was more common in urban locations compared to rural. 12.7% of patients had ICU-specific needs sufficient to be billed by a critical care provider. Total provider costs were significantly higher in patients utilizing the ICU post-operatively, even among uncomplicated patients with routine discharges.

**Conclusion:**

Most patients undergoing elective endovascular UIA repair in the United States are admitted to the ICU postoperatively. Only 12.7% have ICU needs, and these patients are predictable from pre-operative characteristics or peri-operative complications. Reducing ICU use in this subgroup of patients may be an important target to improve healthcare value in this patient population.

## Introduction

Optimizing healthcare resource utilization is essential to help reduce cost, achieve greater system-wide efficiency, and increase healthcare value.^1,2^ Intensive care unit (ICU) beds are an important area of study in the field of healthcare resource utilization. ICU beds serve a vital role in the management of critically ill patients; however, this higher-level care constitutes a limited resource that accrues significantly higher costs.^1,3,4^ The coronavirus disease 2019 pandemic highlighted the importance of appropriate ICU utilization as the scarcity of ICU beds necessitated triage to provide care to those affected by the pandemic.^5–7^ Balancing resource utilization and cost while providing safe care requires an interdisciplinary approach.

Endovascular treatment (EVT) of unruptured intracranial aneurysms (UIAs) has become increasingly safe with growing evidence suggesting that endovascular techniques may have fewer procedural complications and shorter hospital stays compared to open surgical treatment.^8–11^ Technology has increased the number of aneurysms that can be managed with this treatment modality.^8,12,13^ Multiple studies have suggested that routine post-operative ICU admission after EVT for UIAs may be unnecessary and results in higher costs.^14–18^ Further, when ICU-level care is necessary, it is predictable from pre-operative characteristics or related to intraoperative complications.^14,15,18,19^ In the setting of similar safety profiles and cost disparities between floor and ICU care following EVT for UIAs, optimizing the appropriate utilization of post-operative ICU care may be a potential target for interventions seeking to improve healthcare value. However, further information is required regarding the national landscape of post-operative ICU utilization following EVT for UIA.

This study seeks to evaluate trends in ICU utilization among patients undergoing elective EVT of UIA in the United States. We hypothesize that ICU utilization in this patient population remains high nationally, even in the setting of studies indicating ICU-level care may be unnecessary. Further, we hypothesize that patients undergoing EVT for UIA who have a post-operative ICU admission will have significantly higher healthcare costs than those who did not receive ICU care.

## Methods

### Data sources

We performed a retrospective cohort study utilizing the IBM Watson Health MarketScan Database to evaluate the utilization of ICU in patients undergoing elective endovascular repair of unruptured aneurysms between 2005 and 2019. The MarketScan database includes deidentified data on over 43 million patients annually via commercial healthcare claims from employers, health plans, and hospitals, alongside medicare supplemental claims. A separate study of our own institutional data was performed to validate the identification of ICU versus non-ICU admitted patients using accommodation revenue codes. An institutional review board exemption was obtained for both the database portion and for the institutional validation portion of this study.

### Case identification and selection

A combination of diagnosis codes (ICD9: 437.3: cerebral aneurysm, non-ruptured and ICD10: I67.1: cerebral aneurysm, non-ruptured) in combination with CPT procedural codes (61624: transcatheter embolization/occlusion, intracranial/spinal cord) and accommodation revenue codes were utilized to identify our initial cohort. Patients over 18 years old or with a diagnosis code of arteriovenous malformation (ICD9: 747.81 or ICD10: Q28.2) or subarachnoid hemorrhage (ICD9: 747.81 or ICD10: Q28.2) as a primary or secondary diagnosis code were excluded. Patients were also excluded if they had an emergency admission status of the same admission as the procedural code (defined as revenue codes 0450, 0451, 0452, 0456, or 0459). Furthermore, patients with an outpatient emergency department (ED) claim on the same or 1 day prior to admission were excluded. Patients without any admission revenue codes were also excluded. To enable the ascertainment of preoperative comorbidities, patients were required to have 1 year of continuous insurance coverage prior to index admission.

ICU admission after elective endovascular aneurysm treatment (index admission) was identified utilizing one of the accommodation revenue codes specific for intensive care (0200, 0201, 0202, 0203, 0204, 0207, 0208, or 0209). Code 0205 (“intermediate care ICU”) was not included in the ICU group. Specific definitions of codes are provided in the Supplemental Data File. Subgroup analysis was performed to identify those with an ICU revenue code who had critical care billing charges as defined by CPT codes 99291 or 99292.

To validate the reliability of accommodation revenue codes at identifying ICU admissions, 75 sequential patients undergoing elective neurosurgical intervention (equal numbers of consecutive elective endovascular intracranial aneurysm treatment, elective craniotomy for brain tumor, and elective spine surgery) were queried at our institution. Accommodation billing revenue codes identifying level of care post-procedurally were recorded and compared with post-operative level of care identified by individual chart review, and the sensitivity and specificity of the revenue codes for identifying post-operative level of care were calculated (Supplemental Table 1).

### Variables

Demographic and outcomes data were collected including sex at index admission, age at index admission, type of insurance, insurer location (urban vs. rural), Deyo–Charlson index for comorbidity, hypertension, and smoking. Post-procedural complication data on index admission were also collected. Surgical complications included intracerebral hemorrhage, subarachnoid hemorrhage, iatrogenic cerebrovascular infarct/hemorrhage, obstructive hydrocephalus, generalized grand mal seizure and epilepsia partialis continua, hemorrhage/postprocedural hemorrhage, and hematoma following the procedure. Subarachnoid hemorrhage was included as a complication if it was recorded as an admission code beyond the first or second primary diagnosis code. Medical complications included cardiac, pulmonary, renal, and systemic infection. Length of stay and discharge status were also compiled. Specific ICD codes are provided in the supplement. Cost analysis was performed based on total gross payment to all providers. Cost analysis was stratified into uncomplicated and all hospital stays. Uncomplicated stays were defined as those for patients who were discharged home and had a hospital stay ≤ 2 days.

### Statistical analysis

Trends in ICU utilization between 2005 and 2019 were analyzed via the Cochran–Armitage test for trend. Comparisons of ICU utilization by demographics, comorbidities, complications, and discharge disposition were completed using independent t-tests, chi-square testing, and Fischer's exact testing. Total costs of admission were assessed by ICU utilization between those with uncomplicated stays and all patients. Cost analysis was assessed using Wilcoxin-Rank-Sum testing for both total provider and total hospital payments as reported by the database. Annual elective EVT rates in the United States for cost calculations were conservatively estimated from Luther et al., 2020 to be 9705 treatments per year based on 2014 data.^20^ All statistical analyses were carried out at a significance level of *p *< 0.05 using a two-sided test. Statistics analysis was completed using GraphPad Prism 8 and SAS version 9.4.

## Results

### General characteristics and cohort determination

**Figure 1.**
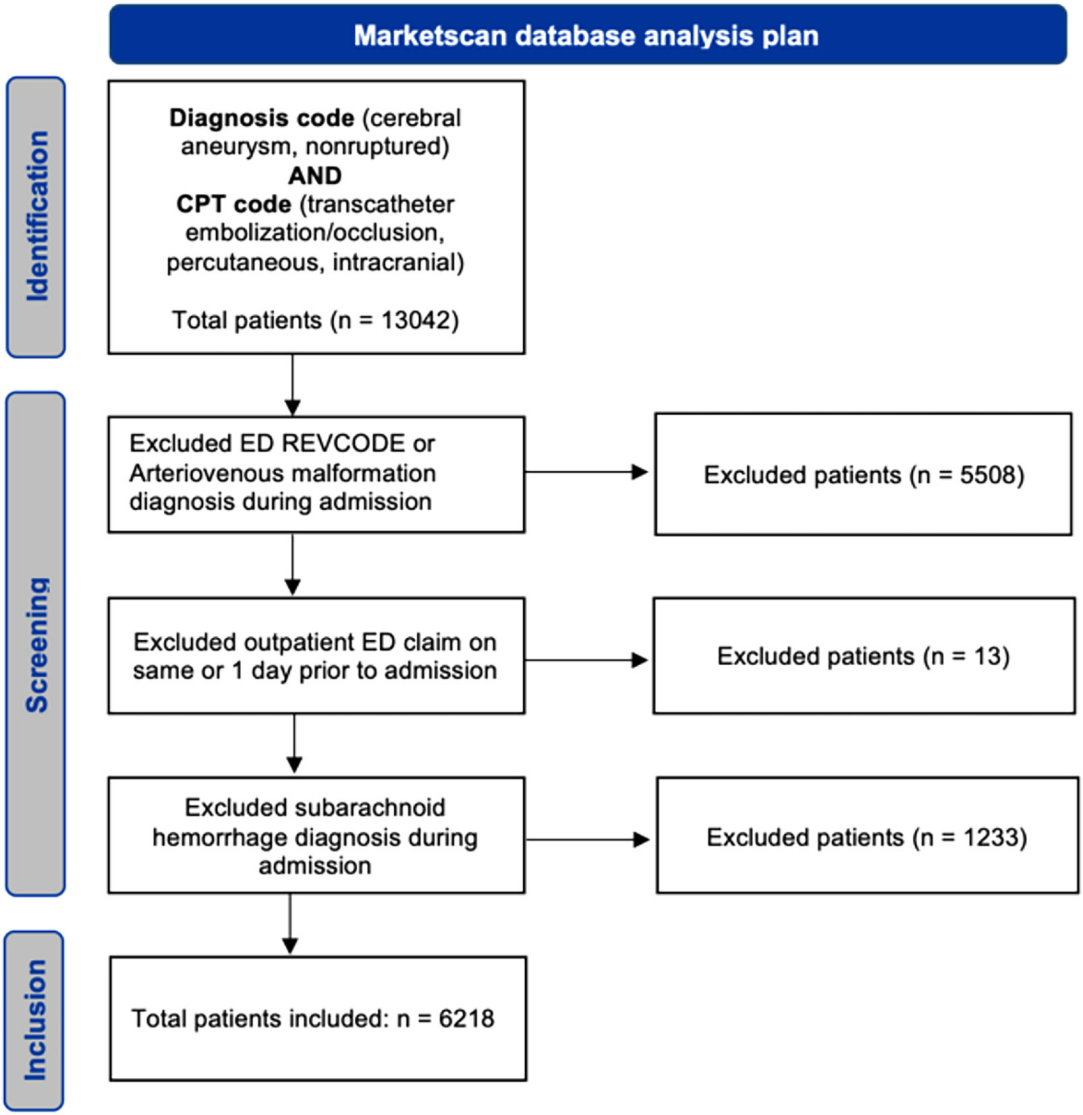
STROBE diagram outlining inclusion and exclusion strategy.

A total of 13,042 patients were included in our initial query by a combination of unruptured aneurysm ICD diagnosis code and endovascular embolization CPT code. Of these, 5508 patients were excluded due to emergency room admission or a diagnosis of arteriovenous malformation during the index admission. 83 patients were excluded due to an outpatient ED claim on the same or 1 day prior to admission or did not have a revenue code. An additional 1233 patients were excluded due to subarachnoid hemorrhage listed as the primary or secondary diagnosis during admission. The final cohort included 6218 patients. The database cohort flow chart is included in Figure 1.

### Institutional validation of accommodation revenue codes for identifying ICU admissions

Our institutional review to assess the reliability of accommodation revenue codes identified 25 consecutive patients via institutional case logs undergoing each of the following procedures: elective EVT of UIA, elective craniotomy for supratentorial brain tumor resection, and elective spine surgery. Consistent with our institutional practices, 23/25 patients undergoing EVT of a UIA, 2/25 patients undergoing elective craniotomy for tumor resection, and 1/25 patients undergoing elective spine surgery were admitted to the ICU postoperatively. In 74 of 75 cases, revenue codes correctly identified their post-operative level of care as identified by chart review. The final validation calculations yielded a 96% sensitivity and 100% specificity of revenue codes for identifying ICU admission post-operatively (Supplemental Table 1).

### Prevalence and trends in ICU utilization

**Figure 2.**
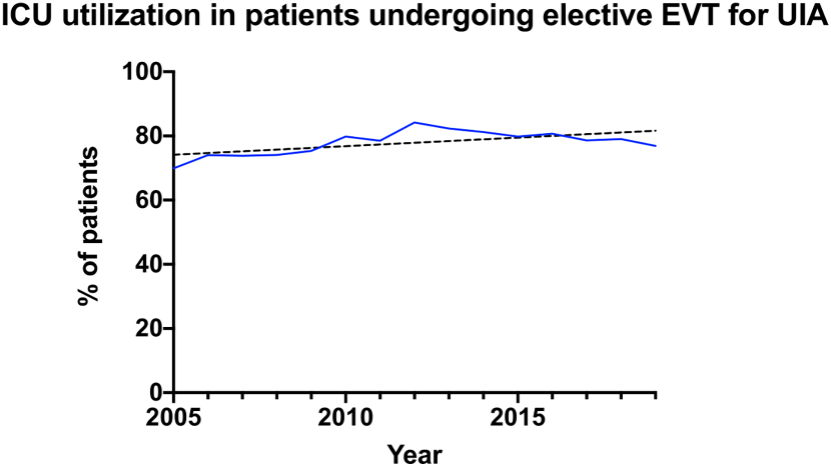
ICU utilization in patients undergoing elective endovascular treatment of unruptured cerebral aneurysms.

Of 6218 total patients, 4890 (78.6%) were admitted to the ICU during their index admission. Annual ICU utilization was consistently near or greater than 75% of all patients treated endovascularly with a slight increase in utilization over the course of the study (Figure 2), with 69.9% of patients admitted to the ICU in 2005 versus 76.9% with ICU admission in 2019. Intensive care utilization differed slightly by insurance status, with patients who were managed post-operatively in the ICU being more likely to have an HMO, CDHP, or HDHP plan (*p *< 0.01). Patients managed in the ICU post-operatively were significantly more likely to live in urban locations (84.0% in ICU group vs. 79.4% in non-ICU group). No significant differences were seen between ICU and non-ICU groups for Charlson Comorbidity index, smoking status, or hypertension (Table 1).

**Table 1.** Demographic and comorbidity information of patients undergoing EVT of UIA

	No ICU (n = 1328)	ICU (n = 4890)	*p*-value
Age	51.9 ± 9.3	51.9 ± 9.2	0.94
Sex			0.41
Male (%)	306 (23.0)	1075 (22.0)	
Female (%)	1022 (77.0)	3815 (78.0)	
Type of insurance			**0**.**007**
EPO (%)	19 (1.4)	57 (1.2)	
HMO (%)	141 (10.6)	587 (12.0)	
POS (%)	139 (10.5)	381 (7.8)	
PPO (%)	828 (62.3)	2982 (60.1)	
Other (%)	155 (11.7)	661 (13.5)	
Patient location			**<0**.**01**
Rural (%)	273 (21.6)	782 (16.0)	
Urban (%)	1055 (79.4)	4108 (84.0)	
Charlson comorbidity index (median, IQR)	2 (1–3)	2 (1–3)	0.88
Smoker	193 (14.5)	802 (16.4)	0.20
Hypertension	693 (52.2)	2504 (51.2)	0.64

Boldface indicates values that are statistically significant.

Patients managed post-operatively in the ICU had a slightly higher number of associated surgical complication codes than those managed on the floor (3.6% vs. 2.3%, *p *= 0.02). Patients admitted to the ICU had significantly more pulmonary complications than those admitted to the floor (2.0% vs. 0.6%, *p *< 0.01). No significant differences were noted in the types of surgical complications between those admitted to the ICU and the floor postoperatively. Lastly, there was no significant difference in discharge status between those who were and were not managed in the ICU postoperatively, with 97.3% of non-ICU patients discharging home versus 96.1% of ICU patients (*p *= 0.18). Likewise, 30-day readmission rates were similar between groups (ICU 6.5% vs. non-ICU 6.1% *p *= 0.34) (Table 2). ICU provider billing as indicated by CPT code 99291 (evaluation and management of critically ill or critically injured patient, first 30–74 min) or 99292 (each additional 30 min) was noted in 12.7% of patients.

**Table 2.** Complications and discharge disposition of patients undergoing elective EVT of UIA

	No ICU (n = 1328)	ICU (n = 4890)	*p*-value
Any complication	31 (2.3)	177 (3.6)	**0**.**02**
Medical complications			
Cardiac	19 (1.4)	69 (1.4)	0.96
Pulmonary	8 (0.6)	100 (2.0)	**<0**.**01**
Infection	0 (0.0)	5 (0.1)	0.12
Renal	5 (0.4)	14 (0.2)	0.59
Surgical complications			
Intraparenchymal hemorrhage	14 (1.0)	68 (1.3)	0.34
Subarachnoid hemorrhage	3 (0.2)	31 (0.7)	0.08
Iatrogenic infarct/hemorrhage	4 (0.3)	34 (0.7)	0.10
Hydrocephalus	9 (0.7)	30 (0.6)	0.80
Post-procedural hematoma	13 (1.0)	40 (0.8)	0.56
Discharge disposition			0.18
Home or self-care	1222 (97.3)	4444 (96.1)	
Home health	16 (1.3)	72 (1.5)	
Rehab	14 (1.1)	91 (2.0)	
Unknown/other	4 (0.3)	19 (0.4)	
30-day readmission rate	81 (6.1%)	320 (6.5%)	0.34

Boldface indicates values that are statistically significant.

### Provider and hospital payments associated with ICU utilization

The median total provider payment was higher in patients admitted to the ICU post-operatively ($40,763.52 vs. $34,764.64, *p *< 0.01). In patients with an uncomplicated hospital stay (defined as discharged home and length of stay ≤ 2 days) median total provider payment was higher in patients admitted to the ICU post-operatively ($39,100.53 vs. $33,708.10, *p *< 0.01).

Assuming 9705 elective EVT annually in the United States,^20^ reduction of ICU utilization from 80% to 20% would save $36,129,851.64 in total provider payments. A reduction of ICU utilization from 80% to 50% would save an estimated $18,064,925.82 in total provider payments.

## Discussion

In our analysis of national private-payer claims data in the United States, we found ICU utilization in patients undergoing elective EVT of UIAs is high nationally, with 78% of patients admitted to the ICU during their index admission between 2005 and 2019. Furthermore, despite the publication of studies demonstrating that routine ICU admission may be unnecessary, as well as continually improving techniques and safety of EVT, the proportion of ICU admissions increased slightly over the study period.^14,15,17^

The total provider payment was higher among those who utilized the ICU not only for all admissions, but also for the subset of those admissions identified as uncomplicated. Additionally, we found that ICU utilization differed by geographic location (urban vs. rural) and insurance type. We hypothesize that urban medical centers may have a more complex case mix as well as a larger ICU capacity to allow for elective admissions. Differences in insurance type are difficult to explain and while statistically significant due to the large sample size, are likely not clinically relevant. In the context of studies indicating that post-operative management on a neurosurgical floor has similar safety outcomes^15,17,19^ and are more cost effective^14^ in patients undergoing EVT for UIA, this high national frequency of ICU utilization in the face of higher costs highlights the potential for improving the value of care for patients undergoing this treatment.^14–17^

### Safety

While the safety of non-ICU admission after EVT cannot be definitively proven through claims data alone, data from our study support multiple previous single-center series suggesting the safety of selective ICU admission.^15,17,19,21^ Richard et al. and Burrows et al. both compared patients undergoing elective endovascular aneurysm treatment who were placed in a step-down unit or an ICU based on physician preference.^15,21^ They noted similar safety and post-operative complication rates with significant cost savings for patients placed in a step-down unit. Kameda-Smith et al. specifically evaluated the timing of complications and noted that nearly all complications occurred intra-operatively or immediately post-operatively.^19^ In a study performed by our group, we noted that only 14% of patients undergoing elective endovascular aneurysm treatment had an ICU-specific need post-operatively, similar to the 12.7% identified in the current study.^18^ Furthermore, the vast majority of ICU needs could be identified in the immediate operative or peri-operative period using risk factors of older age, procedural duration greater than 200 min, and intraoperative complication. Less than 1% of patients (3/382) developing an ICU need more than 6 hours postoperatively.^18^

Our data also noted no significant difference in discharge disposition status among patients admitted to the ICU versus those admitted elsewhere, and no difference in 30-day readmission rates. Our finding that pulmonary complications were significantly higher in the ICU patients was expected given respiratory support is a known ICU-specific need in the postoperative setting. Physician intuition, patient and procedure-specific factors, along with a waiting period in a step-down unit during the postoperative time period, may serve as a meaningful intervention in improving healthcare value.^18^

Providers may be understandably reluctant to change current practices and de-escalate care because they consider neurologic complications to be low-frequency, high-impact events whose avoidance may justify significant cost. Accumulating data from studies such as ours, however, may help inform decisions about balancing safety and cost. Importantly, similar rapid de-escalation has been safely performed in mechanical thrombectomy patients during the COVID pandemic and showed no demonstrable effect on clinical outcomes.^22^ Protocols have also been adopted in the cardiac literature with fast-track strategies showing significantly fewer post-operative length of stay and lower direct costs for elective transcatheter aortic valve replacement.^23,24^

### Cost

Total provider payments were approximately 14% higher in patients admitted to the ICU post-operatively. This difference remained significant even in patients with uncomplicated hospital stays suggesting that cost differences can in part be attributed to charges related to the ICU admission. This finding is not surprising given that ICUs constitute up to 20% of hospitals’ costs.^25^ Graduated reduction in ICU utilization (i.e., 80% to 50% or 20%) within the subgroup of patients studied here may lead to substantial healthcare cost savings, on the order of millions of US dollars. Importantly, this cost analysis does not incorporate savings garnered from more efficient resource allocation and increased availability of ICU beds for treatment of patients with more critical care needs. While our study measured cost through payments made by private insurers, it should be noted that providers or hospitals may not be incentivized to lower ICU utilization if they do not realize these savings themselves. Nonetheless, as the landscape of medical care in the United States shifts away from fee-for-service models toward value-based care, lower and more efficient ICU utilization will be required to make care more cost effective.

## Limitations

Our study is limited by the known shortcomings of administrative data, including coding uncertainty, lack of procedural details, and difficulty measuring functional outcomes.^26^ Previously published single center-studies with more procedural and patient-level detail are complementary and drew similar conclusions at an institutional level as our study does at a national level. Accommodation revenue codes have previously been used to study ICU utilization in this database,^27,28^ and our institutional validation of revenue codes we used to define ICU admissions in a neurosurgical population had a 96% sensitivity and 100% specificity for defining ICU admission (Supplemental Table 1). We could not distinguish between different types of endovascular therapy (coiling, coiling with adjuncts, flow diversion, etc.), or characteristics of aneurysms (size, location, morphology). Intra-operative rupture may be underreported as a surgical complication given our methodology which prioritized excluding patients with ruptured intracranial aneurysms by excluding those with SAH as a primary or secondary diagnosis. Nonetheless, other codes (non-primary SAH codes, ICH, iatrogenic hemorrhage) serve to identify many intraoperative ruptures. Furthermore, under-reporting of intraoperative rupture would not drastically alter our primary results of high ICU utilization and importantly, would likely tend to underestimate ICU utilization within our cohort. Functional outcomes are difficult to measure through claims data. In this study, we used disposition status and 30-day readmission rates as a proxy for post-operative functional status, which has been shown to be correlated, albeit imperfectly, with functional status.^29^ While our study is a large, nationally representative population, it reflects those with private insurance only, and cost considerations may differ for non-private patients. Furthermore, the study focuses on a population within the United States which may not be easily generalized to other countries. Lastly, while some standards for ICU-level care are universal, individual hospital capabilities and practice patterns vary regarding the physician and nursing capabilities of differing levels of care acuity. Specifically, our study cannot assess different institutional ICU admission criteria (i.e., those who admit all patients to the ICU vs. those who selectively admit patients to the ICU) though this data is well studied in previous reports.

## Conclusions

ICU utilization remains high in patients undergoing EVT for UIA despite recent evidence of similar safety and cost-savings for patients who receive non-ICU care postoperatively. Combined with previously published single-center studies with more procedural and patient-level detail, our study supports routine non-ICU management with selective ICU admission after EVT as an important target to conserve resources and improve healthcare value in this patient population.

## Previous presentation

An oral abstract based on this work was presented at the Congress of Neurological Surgeons Annual Meeting in San Francisco in October 2022.

## Supplemental Material

sj-docx-1-ine-10.1177_15910199241233028 - Supplemental material for Nationwide trends in intensive care unit utilization in the elective endovascular treatment of unruptured intracranial aneurysms Supplemental material, sj-docx-1-ine-10.1177_15910199241233028 for Nationwide trends in intensive care unit utilization in the elective endovascular treatment of unruptured intracranial aneurysms by Varun Padmanaban, William J. Benjamin, Austin Cohrs, Francis J. Jareczek, Sprague W. Hazard, Joseph Christopher Zacko, Ephraim W. Church, Scott D. Simon, Kevin M. Cockroft, Douglas L. Leslie and David Andrew Wilkinson in Interventional Neuroradiology
