# Supplementary material for: Nationwide trends in intensive care unit utilization in the elective endovascular treatment of unruptured intracranial aneurysms
Source: Interv Neuroradiol. 2024 Mar 7;32(4):1296–302. doi: 10.1177/15910199241233028 (PMC11569808; doi:10.1177/15910199241233028)
Supplement: Supplementary file 1 [file sj-docx-1-ine-10.1177_15910199241233028.docx]

Supplemental Data:

**Elective unruptured aneurysm treatment, endovascular**

1. Coding inclusion:
   a. **Diagnosis code:** Cerebral aneurysm, nonruptured
      i. ICD9 – 437.3
      ii. ICD10 – I67.1
   b. **CPT code:**
      i. 61624 - transcatheter embolization/occlusion, percutaneous, intracranial/spinal cord
2. Coding exclusion
   a. Patient $\leq$ 18 years old
   b. **Diagnosis code:** Arteriovenous malformation of cerebral vessels
      i. ICD9 – 747.81
      ii. ICD10 – Q28.2
   c. Emergency admission status
      i. REVCODE (0450, 0451, 0452, 0456, 0459)

| **REVCODE** | **Definition** |
| --- | --- |
| 0450 | Emergency room – general classification |
| 0451 | Emergency room – emtala emergency medical screening |
| 0452 | Emergency room – ER beyond emtala screening |
| 0456 | Emergency room – urgent care |
| 0459 | Emergency room – other |

**Defining ICU admission**

1. Coding inclusion:

| **REVCODE** | **Definition** |
| --- | --- |
| 0200 | Intensive care – general classification |
| 0201 | Intensive care – surgical |
| 0202 | Intensive care – medical |
| 0203 | Intensive care – pediatric |
| 0204 | Intensive care – psychiatric |
| 0207 | Intensive care – burn |
| 0208 | Intensive care – trauma |
| 0209 | Intensive care – other intensive care |

*Subgroup analysis*: ICU REVCODE with critical care billing as defined by CPT code 99291 (first 30-74 minutes of critical care) or 99292 (each additional 30 minutes of critical care) on index admission

**Demographics**

- Sex (at index event)
- Age (at index event)
- Type of insurance
- Insurer location (urban versus rural)
- Deyo-Charlson index for comorbidity (require 1 year of continuous coverage prior to index event)
- Hypertension
  - ICD9 – 401.0, 401.0, 401.9
  - ICD10 – I10
- Smoking
  - ICD9 – 305.1
  - ICD10 – F17.2 (nicotine dependence) or Z87.8 (history of nicotine dependence)
- Length of stay (days)

**Post-procedural complication data (on index admission date ONLY)**

- Surgical
  - Intracerebral hemorrhage (ICD9 431; ICD10 G97.51)
  - Subarachnoid hemorrhage (ICD9 430; ICD10 I60.9)
  - Iatrogenic cerebrovascular infarction/hemorrhage (ICD9 & 10 997.02)
  - Obstructive hydrocephalus (ICD9 331.4; ICD10 G91.1 )
  - Generalized grand mal status and epilepsia partialis continua (ICD9 345.4, 345.7; ICD10 G40.309; 345.71)
  - Hemorrhage/postprocedural hemorrhage and hematoma following other procedure (ICD9 998.11, ICD10 I97.618, I97.62, I97.610)
- Medical
  - Cardiac including arrhythmia, acute MI, CHF, cardiac arrest (ICD9 997.1, 427.8, 427.9, 428.0, 428.9, 410, 427.5; ICD10 I49.9, I21.9; I50.2, I46)
  - Pulmonary complications including acute respiratory distress, respiratory failure, acute respiratory arrest (ICD9 518.81, 518.82, 799.1; ICD10 J96.00, J96.92, R09.2)
  - Systemic infection including bacteremia, septicemia and SIRS (ICD9 790.7, 038, 995.92, 995.94; ICD10 R78.81; A41.9, R65.10)
  - Acute renal failure including nontraumatic acute tubular, cortical and medullary necrosis and nephropathy not otherwise specified (ICD9 584, 584.5, 584.6, 584.7, 584.8, 584.9, 583; ICD10 N17.0, N17.1, N17.2, N17.8, N17.9, N99.0)

**Discharge status**

- In-hospital mortality: DSTATUS 20 (*not available beginning in 2016*)
- Discharge to home: DSTATUS 1
- Discharge to short-term hospital (DSTATUS 2)
- Discharge/transfer to SNF (DSTATUS 3)
- Discharge/transfer to ICF (DSTATUS 4)
- Discharge/transfer to other facility (DSTATUS 5)
- Discharge/transfer to home health service (DSTATUS6)
- Transfer to inpatient rehab (DSTATUS 62)
- Transfer to LTCH (DSTATUS 63)
- Transfer to nursing facility Medicaid (DSTATUS 64)

**Supplemental Table 1**: Institutional validation of REVCODES for ICU admissions for unruptured intracranial aneurysms.

|  | ICU Admission | No ICU Admission | Total |
| --- | --- | --- | --- |
| ICU admission | 25 | 0 | 25 |
| No ICU admission | 1 | 49 | 50 |
| Total | 26 | 49 | 75 |

Sensitivity – 96%

Specificity – 100%
